# Clinical effectiveness of spinal cord stimulation in managing refractory upper-extremity pain

**DOI:** 10.1007/s10143-025-04123-7

**Published:** 2026-02-20

**Authors:** Yanisa Ingkapassakorn, Songrit Vuttipongkul, Bunpot Sitthinamsuwan, Sukunya Jirachaipitak, Pramote Euasobhon, Nantthasorn Zinboonyahgoon, Sarun Nunta-aree

**Affiliations:** 1 Division of Neurosurgery, Department of Surgery, Faculty of Medicine Siriraj Hospital, Mahidol University, 2 Wang Lang Road, Bangkok Noi, Bangkok, 10700 Thailand; 2 Department of Anesthesiology, Faculty of Medicine Siriraj Hospital, Mahidol University, Bangkok, Thailand; 3 Siriraj Pain Management Unit, Faculty of Medicine Siriraj Hospital, Mahidol University, Bangkok, Thailand

**Keywords:** Refractory pain, Spinal cord stimulation, Upper extremity, Upper limb

## Abstract

Spinal cord stimulation (SCS) is an effective therapy for intractable pain, but it is used less frequently for upper extremity than for lower limb pain. Evidence supporting SCS for the upper extremity remains limited. This study evaluated the efficacy of SCS for severe refractory upper extremity pain. Thirteen patients with refractory upper extremity pain underwent a trial of SCS. Eleven patients, who achieved marked pain relief during the trial, proceeded to permanent implantation. We collected clinical characteristics and outcomes, including numeric pain rating scale (NPRS) measurements. We then compared pain reduction among subgroups: neural versus non-neural lesions, and complex regional pain syndrome (CRPS) versus non-CRPS. In the 11 implanted patients, the mean NPRS score decreased from 9.5 of 10 preoperatively to 3.6 of 10 postoperatively (*p* < 0.001). Analysis showed significant pain improvement in each subgroup (*p* < 0.05). At last follow-up, the NPRS scores remained below baseline levels in 10 of 11 patients. However, the magnitude of the NPRS score reduction did not differ significantly between neural and non-neural lesions (*p* = 0.350), or CRPS and non-CRPS (*p* = 0.245). One participant with CRPS type 2 experienced treatment failure during long-term follow-up. This study demonstrated that SCS effectively alleviates refractory upper extremity pain caused by various etiologies. No single pain etiology, including CRPS, conferred a superior response, suggesting broad potential benefits of SCS in these patients.

## Introduction

 Chronic pain is defined as pain that persists or recurs for longer than 3 months [1]. It is a complex disease process and a major source of disability and economic burden [2, 3]. Various conditions commonly underlie chronic pain, including chronic neuropathic pain, complex regional pain syndrome (CRPS), failed back surgery syndrome, failed neck surgery syndrome, chronic cancer-related pain, inflammatory arthritis, fibromyalgia, and chronic migraine [4–14]. Managing chronic pain requires a multimodal approach, including pharmacological therapy, interventional procedures, physical rehabilitation, lifestyle modifications, psychological therapy, neuromodulation, and surgery [15, 16].

In addition to the commonly encountered back and lower limb pain in failed back surgery syndrome, upper extremity pain also significantly reduces quality of life. Severe shoulder, arm, forearm, hand, or finger pain can markedly impair daily activities. Chronic upper limb pain may be caused by posttraumatic or postsurgical neuropathic pain, failed neck surgery syndrome, postherpetic neuralgia, CRPS, phantom limb pain, and peripheral limb ischemia [17–27].

Because most patients with intractable upper limb pain retain adequate limb function, neuromodulation can achieve pain control while preserving functionality [28–31]. Several neuromodulatory techniques have been proposed, including spinal cord stimulation (SCS), peripheral nerve stimulation, and dorsal root ganglion stimulation [32–42]. SCS is a well-established therapy for chronic refractory upper extremity pain [43–45]. This procedure involves placing epidural leads on the posterior aspect of the spinal cord. Stimulation of the dorsal column pathways transforms painful sensations into paresthesias, thereby alleviating pain [46].

Although numerous studies have shown the efficacy of thoracic and lumbar SCS [47–50], evidence supporting SCS for upper extremity pain relief remains limited. Therefore, we conducted this study to evaluate the effectiveness of SCS, primarily cervical SCS, in managing chronic intractable upper extremity pain.

## Materials and methods

### Patient population

All patients who underwent SCS for refractory pain at our institute between January 2016 and October 2024 were identified from medical and operative records. This study included those individuals with chronic, intractable upper extremity pain of various etiologies who received SCS. Patients who underwent SCS exclusively for trunk or lower limb pain were excluded. No patient presented with isolated neck pain or combined neck and upper extremity pain. All included subjects received SCS at the cervical or lower cervical to upper thoracic spinal levels.

### Preoperative assessment

At our institute, SCS was considered for intractable pain in patients who had not responded to medical therapy, definitive surgical treatment for underlying conditions, intensive physical therapy, or invasive pain interventions. Additional prerequisites included concurrent psychiatric management and failure of noninvasive electrical stimulation. Such stimulation encompassed transcutaneous electrical nerve stimulation or transcranial magnetic stimulation.

Before the trial, each candidate was rigorously evaluated by pain physicians, psychiatrists, and neurosurgeons. Candidates deemed unsuitable for SCS based on clinical or psychiatric criteria were excluded. Common exclusion reasons included poorly controlled psychiatric illness, multifocal or ill-defined pain not optimally addressed by SCS, unrealistic expectations of pain relief, and patient decision against proceeding with treatment. In the absence of contraindications, an SCS trial was scheduled.

### Operative procedures

#### Spinal cord stimulator trial

**Fig. 1:**
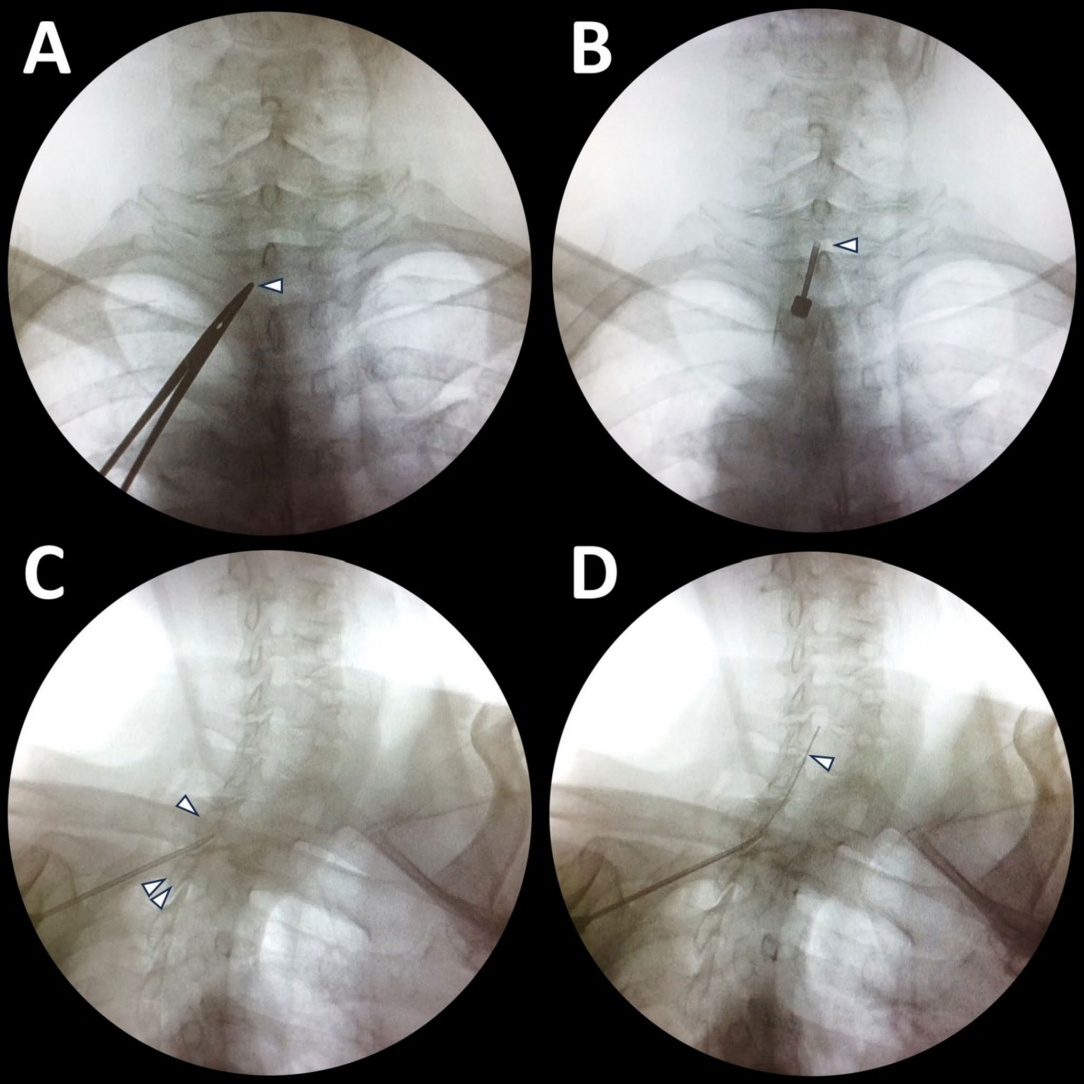
Fluoroscopic guidance for epidural access during cervical SCS (prone). **(A)** Skin entry site (arrowhead), anteroposterior (AP) view. **(B)** Insertion of a 14-gauge Tuohy needle toward the target interlaminar space (arrowhead), AP view. **(C)** Needle passing the interlaminar window between the upper (single arrowhead) and lower (double arrowheads) laminae, oblique view. **(D)** Guide-wire (arrowhead) freely advanced into the epidural space

In all SCS procedures at our institute, we used devices from Medtronic (Fridley, MN, USA). The trial was typically conducted with the patient awake and prone in the operating theater, unless open surgery with direct placement of trial electrodes was required. Target vertebral levels were selected based on each patient’s pain distribution. After sterile preparation, we identified both the skin and epidural entry points using intraoperative fluoroscopy (Fig. 1A and B). For upper limb pain, the common entry site was at the upper thoracic region. Local anesthetic was injected into the skin, fascia, and paraspinal muscles. A 14-gauge Tuohy epidural needle (12 cm) was introduced through the interlaminar space (Fig. 1C). Epidural entry was confirmed via the loss-of-resistance technique with air, after which a guidewire was advanced under fluoroscopic guidance to verify correct placement (Fig. 1D). The guidewire was then removed.

**Fig. 2:**
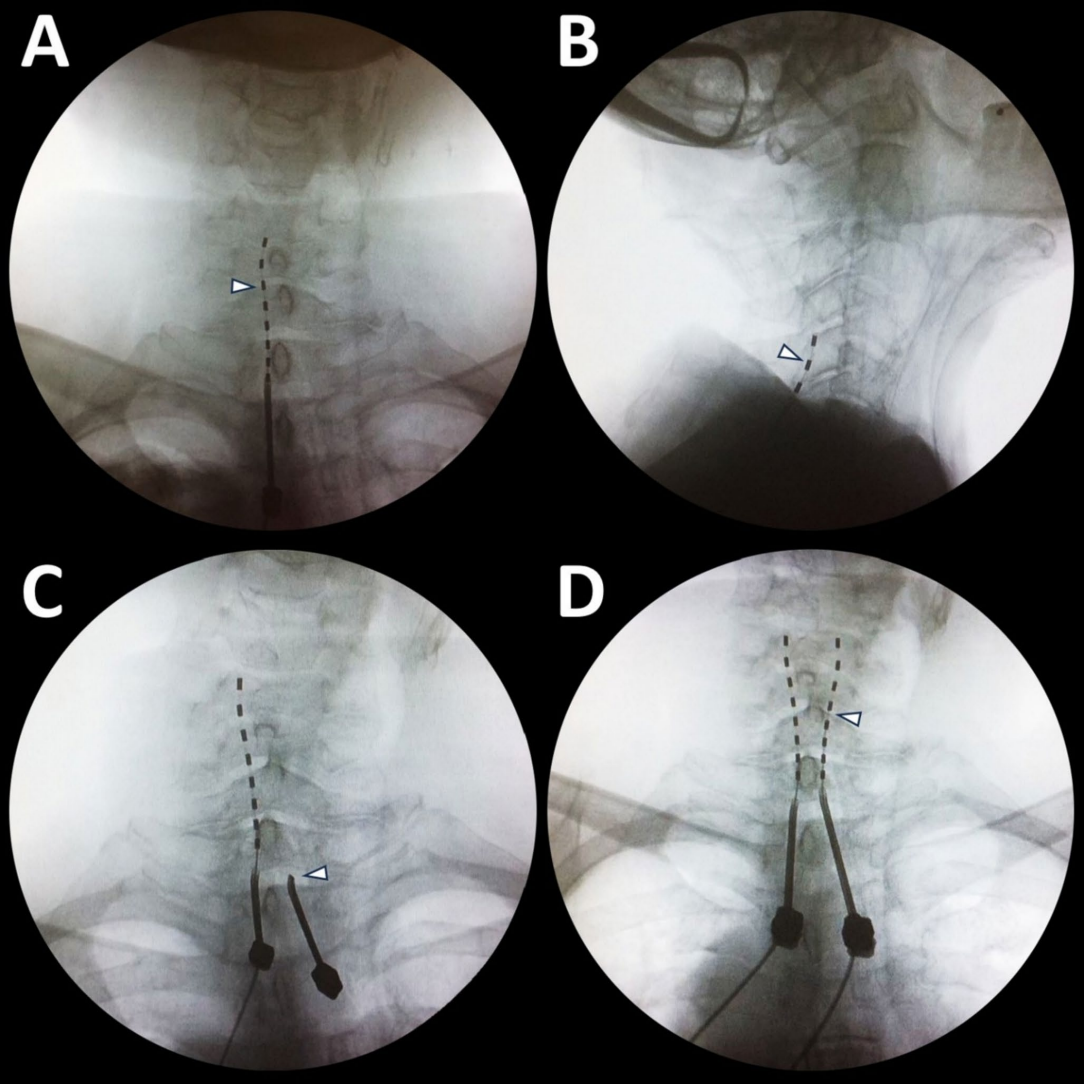
Percutaneous placement of two epidural leads (prone). **(A)** First lead (arrowhead) introduced through a Tuohy needle, AP view. **(B)** Lead confirmed posterior to the spinal cord (arrowhead), lateral view. **(C)** Second Tuohy needle positioned in the interlaminar space (arrowhead), AP view. **(D)** Second lead (arrowhead) advanced to the target vertebral levels, AP view

A percutaneous trial electrode with 8 contacts (60 cm) was next introduced through the epidural needle and navigated rostrally to the intended spinal level. Fluoroscopy in anteroposterior and lateral views (Fig. 2A and B) confirmed correct positioning. If the electrode appeared too far anterior, we revised its placement. Once satisfactory positioning was achieved, the electrode was connected to an external neurostimulator. We adjusted stimulation parameters—active contacts, current intensity, frequency, and pulse width—to produce paresthesia covering the painful region. The patient verbally confirmed complete coverage of the affected area. If necessary, an additional trial electrode was inserted (Fig. 2C and D). Finally, the externalized electrodes were anchored to the skin, secured with sterile waterproof dressings, and connected to the external neurostimulator.

#### Stimulus parameter setting and stimulation during the trial period

**Fig. 3:**
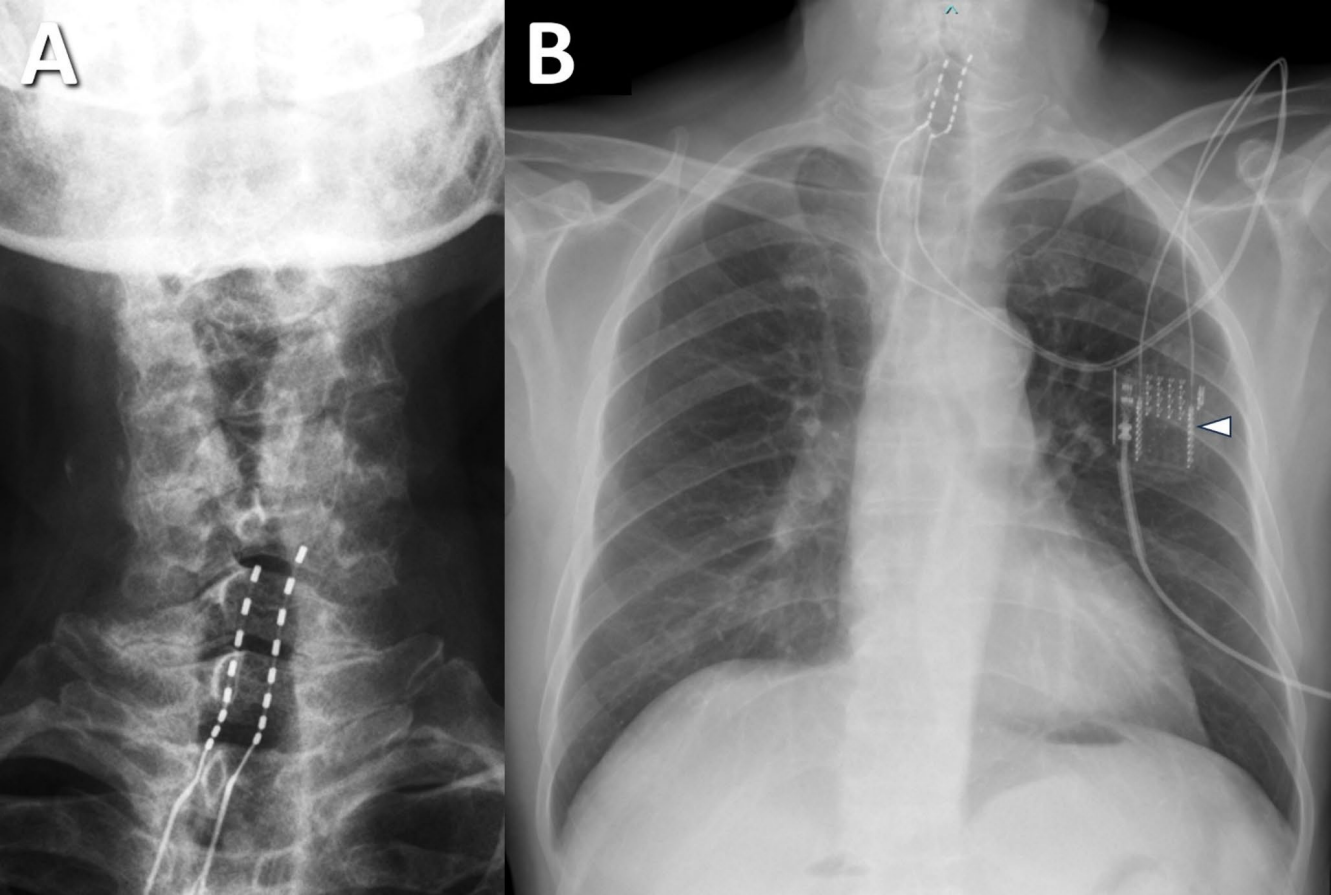
Post-trial radiographs demonstrating electrode positions. **(A)** Cervical spine, AP view, showing lead contacts relative to vertebral levels. **(B)** Chest radiograph showing leads at the lower cervical level and connection to an external neurostimulator (arrowhead)

After the trial procedure, postoperative radiography was performed to confirm the exact vertebral levels of the electrode contacts (Fig. 3). We then programmed stimulation parameters on the external neurostimulator, creating multiple settings to optimize pain coverage. Each patient could switch among these settings to find the most effective stimulation pattern. Once programming was complete, patients were usually discharged with the neurostimulator for a 7‒14-day trial period. They were encouraged to maintain normal daily activities while avoiding immobilization or excessive rest. After the trial, patients returned to our outpatient clinic for pain assessment, and the trial electrodes were removed. Individuals who achieved at least 50% reduction in pain intensity relative to baseline were deemed suitable for permanent SCS implantation. We also documented which electrode contacts conferred the greatest pain relief. Patients who did not meet the 50% threshold did not proceed to permanent implantation.

#### SCS device implantation

Permanent percutaneous electrode placement followed the same technique as the trial procedure. Once the electrodes were positioned properly, their distal ends were tunneled subcutaneously and connected to an implantable pulse generator placed in a subcutaneous pocket in the unilateral lower back region. In rare cases, extensive epidural fibrosis after laminectomy precluded a percutaneous approach, necessitating open surgery with paddle electrode placement.

### Outcome assessment

Pain intensity was evaluated using the numeric pain rating scale (NPRS). We compared pain scores between the preoperative baseline and the final follow-up after SCS. The percentage of pain reduction was determined by subtracting the NPRS score recorded after SCS from the preoperative baseline score, then dividing by the baseline value. We also compared outcomes between subgroups. These subgroups included patients with pain originating from neural structures (neural-lesion group) vs. those with pain unrelated to neural structures (non-neural-lesion group), and those diagnosed with CRPS (CRPS group) vs. those without CRPS (non-CRPS group).

### Outcomes and hypothesis

The primary outcome of the research was the change in pain intensity evaluated by NPRS from the preoperative baseline to the last follow-up. Secondary outcomes included subgroup comparisons, durability of response, and complications. We hypothesized that SCS could be helpful in long-term pain intensity reduction, good durability of response, and low complication rate. Furthermore, subgroup analysis of the outcomes might be useful in the identification of good surgical candidates for the SCS procedure.

### Statistical analysis

Data were analyzed using IBM SPSS Statistics, version 29 (IBM Corp, Armonk, NY, USA). Descriptive statistical analysis was emphasized in this study. Numerical data are presented as means and standard deviations, and categorical data are reported as counts with percentages. Fisher’s exact test was used to compare 2 independent categorical variables. The strength of association between categorical variables was revealed by odds ratio and a 95% confidence interval. The paired *t*-test was used to evaluate 2 dependent numerical variables, and the independent *t*-test was employed for comparing 2 independent numerical variables. A *P* value of less than 0.05 was considered statistically significant.

Effect size between two compared independent groups was analyzed using Cohen’s d for the independent sample *t*-test. Effect size between preoperative and postoperative NPRS scores in a dependent group was investigated by Cohen’s d for the paired *t*-test. The effect magnitudes of Cohen’s d were described as follows: d = 0–0.19 was interpreted as very small effect size; d = 0.2 as small effect size; d = 0.5 as medium effect size; d = 0.8 as large effect size; and d > 1 as very large effect size.

## Results

### Demographic characteristics

Thirteen patients underwent an SCS trial for refractory upper limb pain during the study period. Two patients with neuropathic pain from cervical syringomyelia related to Chiari I malformation did not respond to the trial. The remaining 11 achieved more than 50% pain reduction and proceeded to SCS device implantation; these 11 were included in the final analysis (Table 1). Their mean age was 45.7 ± 13.6 years (range, 21‒63). Four (36.4%) were male, and 7 (63.6%) were female. The most common pain etiology was CRPS (5 patients, 45.4%), followed by tumor-related neuropathic pain involving spinal cord or nerve roots (2 patients, 18.2%) and cervical root avulsion from brachial plexus injury (2 patients, 18.2%). One patient had peripheral arterial occlusion (9.1%), and another had cervical syringomyelia from Chiari I malformation (9.1%). The mean duration of symptoms before SCS was 47.9 ± 29.2 months (range, 4‒94). Subgroup division is demonstrated in Table 2.

**Table 1:** Baseline characteristics and surgical outcomes of 11 patients who underwent cervical spinal cord stimulation for refractory upper-extremity pain

Patient	Sex and age (y)	Cause of pain	Location of primary lesion	Pain duration before SCS (m)	Pain area	Failed treatment before SCS	Type and spinal level of electrode placement	Preop NPRS score	Postop NPRS score	Reduction in NPRS score	Pain reduction (%)	Postop follow-up (m)	Remarks
1	F 60	Cervical cord ependymoma (post-tumor resection)	Spinal cord	41	Left C8-T1 dermatomes and anterior chest wall	AED, opioid, duloxetine, TMS, IV lidocaine infusion	Paddle, C7-T2	7	3	4	57.1	64	Intermittent loss of stimulation effect related to neck position
2	F 44	Cervical syringomyelia (Chiari I malformation)	Spinal cord	91	Left C5-T1 dermatomes	Posterior cranial fossa decompression, AED, fluoxetine, opioid, TCA,	Percutaneous, both leads at C3-C6	10	4	6	60	15	Rarely unwanted leg stimulation
3	M 35	Brachial plexus avulsion injury	Spinal nerve root	15	Left C6-C8 dermatomes	DREZL, AED, TCA, stellate ganglion block	Paddle, C5-C7	10	2	8	80	38	
4	M 45	Brachial plexus avulsion injury	Spinal nerve root	66	Left C8-T1 dermatomes	AED, opioid, TCA, IV lidocaine infusion, interscalene nerve block, stellate ganglion block, interfascial plane block	Percutaneous, first lead at C5-C7, second lead at C6-T1	10	3	7	70	34	
5	F 54	Cervical nerve root schwannoma (post-tumor resection)	Spinal nerve root	35	Left axilla, medial arm, and anterior chest wall	Reoperation with lysis of nerve root adhesion, AED, opioid, TCA, PT, TENS	Percutaneous, both leads at T1-T3	10	3	7	70	12	Stimulation did not cover the entire anterior chest wall
6	F 48	CRPS type 2 (brachial plexitis)	Peripheral nerve	64	Left upper extremity	AED, NSAIDs, opioid, TCA, cervical ESI	Percutaneous, both leads at C5-C7	9	4	5	55.6	53	Intermittent loss of stimulation effect related to neck position
7	F 63	CRPS type 2 (ulnar neuropathy at the elbow)	Peripheral nerve	49	Left medial arm and forearm	Ulnar nerve transposition, AED, opioid, NSAIDs, sertraline, TCA, ulnar nerve block	Percutaneous, both leads at C5-T1	10	10	0	0	27	Loss of efficacy, removal of SCS device at 16 m after implantation
8	M 62	Ischemic pain due to arterial occlusion (EGPA)	Hand	4	Bilateral hands and fingers	Fentanyl patch, morphine syrup, opioid, prednisolone, TCA, stellate ganglion block	Paddle, C3-C6	9	0	9	100	73	Preop: progressive gangrene of several fingers with severe intractable ischemic pain Postop: dramatic pain relief with marked improvement of distal blood flow
9	F 41	CRPS type 2 (post-tumor resection and RT for desmoid fibromatosis)	Shoulder	94	Left shoulder and lateral arm, medial forearm and hand	AED, morphine, NSAIDs, opioid, TCA, tizanidine, stellate ganglion block	Percutaneous, first lead at C5-C7, second lead at C6-T1	9	5	4	44.4	68	
10	M 21	CRPS type 2 (venous malformation)	Hand	47	Hypothenar area of the left hand	Intralesional bleomycin injections, NSAIDs, opioid, stellate ganglion blocks, AED, TCA, ulnar nerve blocks	Percutaneous, first lead at C4-C6, second lead at C5-C7	10	5	5	50	79	Pain at IPG site which required relocation of IPG at 41 m after SCS
11	F 30	CRPS type 1 (minor trauma)	Hand	21	Left hand and forearm, hand swelling and discoloration with chronic ulcer of the hand	AED, duloxetine, NSAIDs, opioid, prednisolone, hydrotherapy, immobilization, PMS, PT, shock wave therapy, ultrasound therapy, TENS, TMS, stellate ganglion block	Percutaneous, first lead at C2-C4, second lead at C3-C5	10	0	10	100	71	Preop: swollen and discolored left hand with an unhealed chronic ulcer of the hand Postop: marked pain relief with complete resolution of swelling, discoloration, and the ulcer. Premature battery depletion at 1 y after SCS which required a new IPG replacement.

*AED* antiepileptic drug, *C* cervical, *CRPS* complex regional pain syndrome, *DREZL* dorsal root entry zone lesioning; EGPA, eosinophilic granulomatosis with polyangiitis (Churg–Strauss syndrome); ESI, epidural steroid injection, *F* female, *IPG* implantable pulse generator, *IV* intravenous, *M* male, *m* month, *NPRS* numeric pain rating scale, *NSAIDs* non-steroidal anti-inflammatory drugs, *PMS* peripheral magnetic stimulation, *Postop* postoperative, *Preop* preoperative, *PT* physical therapy, *RT* radiation therapy, *SCS* spinal cord stimulation, *T* thoracic, *TCA* tricyclic antidepressant; *TENS* transcutaneous magnetic stimulation, *TMS* transcranial magnetic stimulation, *y* year

**Table 2:** Subgroup division based on involvement of the neural structures or diagnosis of complex regional pain syndrome (CRPS)

Patient	Primary location of pain origin	Diagnosis	Subgroup stratification			
			Involvement of the neural structures		Diagnosis of CRPS	
			Neural-lesion ^a^ (n = 7)	Non-neural-lesion ^b^ (n = 4)	CRPS ^c^ (n = 5)	Non-CRPS ^d^ (n = 6)
1	Spinal cord	Cervical cord tumor	+			+
2	Spinal cord	Cervical syringomyelia	+			+
3	Spinal nerve root	Brachial plexus avulsion injury	+			+
4	Spinal nerve root	Brachial plexus avulsion injury	+			+
5	Spinal nerve root	Cervical nerve root tumor	+			+
6	Peripheral nerve	CRPS type 2: brachial plexitis	+		+	
7	Peripheral nerve	CRPS type 2: ulnar neuropathy	+		+	
8	Hand	Ischemic limb pain		+		+
9	Shoulder	CRPS type 2: soft tissue tumor		+	+	
10	Hand	CRPS type 2: venous malformation		+	+	
11	Hand	CRPS type 1: minor trauma		+	+	

+, present in an individual group; *CRPS* complex regional pain syndrome; *n* number of patients

^a^ Neural-lesion group is defined as the group of patients with potential cause of pain primarily originating from lesions in the neural structures, such as the peripheral nerve, spinal nerve root, or spinal cord. This group included Patients 1-7

^b^ Non-neural-lesion group is defined as the group of patients with potential cause of pain not primarily originating from lesions in the neural structures. This group included Patients 8-11

^c^ CRPS group is defined as the group of patients with a diagnosis of CRPS. This group included Patients 6, 7, and 9-11

^d^ Non-CRPS group is defined as the group of patients without a diagnosis of CRPS. This group included Patients 1-5 and 8

### SCS trial and device implantation

Among the 11 patients, 2 (Patients 1 and 3) required open surgery for trial electrode placement. Patient 1 had a history of cervical laminectomy and resection of a spinal cord ependymoma. Post-surgical epidural fibrosis prevented percutaneous electrode placement, prompting an open approach. Patient 3 had brachial plexus root avulsion pain and underwent dorsal root entry zone lesioning via cervical hemilaminectomy several years before SCS. Recurrent, intractable pain led to open surgery for trial electrode placement. The remaining 9 patients underwent the standard percutaneous approach.

Following a successful trial in all 11 patients, 8 received permanent percutaneous electrodes, while 3 received paddle electrodes. The mean postoperative follow-up was 48.5 ± 24.3 months (range, 12‒79).

### Surgical outcomes and complications

Overall, patients experienced a significant reduction in pain during the follow-up period (*p* < 0.001). Analysis showed that all subgroups—neural-lesion, non-neural-lesion, CRPS, and non-CRPS—also achieved significant postoperative pain relief (Table 3). In the same manner, the effect size analysis revealed a very large magnitude of difference between the preoperative and postoperative NPRS scores in overall patients and in the individual subgroup. These findings indicated that SCS yielded clinically meaningful upper-extremity pain reduction.

**Table 3:** Pain outcomes before and after spinal cord stimulation and subgroup analyses

	Preop vs postop NPRS scores		Postop pain reduction					
	Preop NPRS score,mean ± SD	Postop NPRS score,mean ± SD	Reduction in NPRS score ^c^,mean ± SD	Percentage pain reduction (%) ^d^,mean ± SD	*P* value	Effect size for the paired *t*-test		
						Cohen's d	95% CI	Magnitude
Overall (n = 11)	9.5 ± 0.9	3.6 ± 2.7	5.9 ± 2.8	62.5 ± 27.8	< 0.001^a^	2.932^b^	1.697 to 4.166	Very large
Neural- and non-neural-lesion groups								
Neural-lesion group ^e^ (n = 7)	9.4 ± 1.1	4.1 ± 2.7	5.3 ± 2.7	56.1 ± 26.2	0.002^a^	2.571^b^	1.416 to 3.726	Very large
Non-neural-lesion group ^f^ (n = 4)	9.5 ± 0.5	2.5 ± 2.5	7 ± 2.6	73.6 ± 26.5	0.018^a^	3.883^b^	2.418 to 5.348	Very large
CRPS and non-CRPS groups								
CRPS group ^g^ (n = 5)	9.6 ± 0.5	4.8 ± 3.6	4.8 ± 3.6	50 ± 35.6	0.039^a^	1.868^b^	0.851 to 2.884	Very large
Non-CRPS group ^h^ (n = 6)	9.3 ± 1.2	2.5 ± 1.4	6.8 ± 1.7	72.9 ± 15.6	< 0.001^a^	5.215^b^	3.396 to 7.035	Very large

CI, confidence interval; CRPS, complex regional pain syndrome; n, number of patients; NPRS, numeric pain rating scale; postop, postoperative; preop; preoperative; SCS, spinal cord stimulation; SD, standard deviation

^a^ Indicates a statistically significant difference between the preoperative and postoperative NPRS scores in the individual group.

^b^ Indicates a very large magnitude of difference between the preoperative and postoperative NPRS scores in the individual group.

^c^ Reduction in the NPRS score is defined as the difference between the preoperative and postoperative NPRS scores.

^d^ Percentage pain reduction is defined as the percentage reduction in the NPRS score after SCS.

^e^ Neural-lesion group is defined as the group of patients with potential cause of pain primarily originating from lesions in the neural structures, such as the peripheral nerve, spinal nerve root, or spinal cord. This group included Patients 1-7.

^f^ Non-neural-lesion group is defined as the group of patients with potential cause of pain not primarily originating from lesions in the neural structures. This group included Patients 8-11.

^g^ CRPS group is defined as the group of patients with a diagnosis of CRPS. This group included Patients 6, 7, and 9-11.

^h^ Non-CRPS group is defined as the group of patients without a diagnosis of CRPS. This group included Patients 1-5 and 8.

Comparisons between these subgroups demonstrated no statistically significant differences in clinical variables or outcomes (Table 4 and Table 5). However, in a comparison of postoperative NPRS score and percentage pain reduction between the CRPS and non-CRPS groups using effect size analysis (Table 5), the non-CRPS group had a lower postoperative NPRS score and greater percentage pain reduction with a large magnitude of difference when compared with the counterpart.

**Table 4:** Comparison of clinical variables and pain outcomes between neural-lesion and non-neural-lesion groups

		Overall (n = 11)	Neural- vs non-neural-lesion groups					
			Neural-lesion groups ^c^(n = 7)	Non-neural-lesion group ^d^(n = 4)	*p-*value	Effect size for independent sample *t*-test		
						Cohen’s d	95% CI	Magnitude
Age (y), mean ± SD		45.7 ± 13.6	49.9 ± 9.8	38.5 ± 15.3	0.196	0.957^b^	-0.349 to 2.262	Large
Pain duration before SCS (m), mean ± SD		47.9 ± 29.2	51.6 ± 24.7	41.5 ± 34	0.530	0.359	-0.881 to 1.599	Small
Preop NPRS score, mean ± SD		9.5 ± 0.9	9.4 ± 1.1	9.5 ± 0.5	0.910	0.106	-1.123 to 1.335	Very small
Postop NPRS score, mean ± SD		3.6 ± 2.7	4.1 ± 2.7	2.5 ± 2.5	0.365	0.607	-0.653 to 1.867	Medium
Reduction in NPRS score ^e^, mean ± SD		5.9 ± 2.8	5.3 ± 2.7	7 ± 2.6	0.350	0.637	-0.626 to 1.901	Medium
Percentage pain reduction (%) ^f^, mean ± SD		62.5 ± 27.8	56.1 ± 26.2	73.6 ± 26.5	0.340	0.665	-0.601 to 1.932	Medium
Follow-up (m), mean ± SD		48.6 ± 24.3	34.7 ± 19	72.8 ± 4	0.004^a^	2.429^b^	0.765 to 4.093	Very large
						Odds ratio	95% CI	
Sex, n (%)	Male	4 (36.4)	2 (28.6)	2 (50)	0.576	1.000		
	Female	7 (63.6)	5 (71.4)	2 (50)		2.500	0.194 to 32.195	

CI, confidence interval; m, month; n, number of patients; NPRS, numeric pain rating scale; SCS, spinal cord stimulation; SD, standard deviation; y, year

^a^ Indicates statistically significant difference of data between both groups.

^b^ Indicates a large or very large magnitude of difference of the individual variable between both groups

^c^ Neural-lesion group is defined as the group of patients with potential cause of pain primarily originating from lesions in the neural structures, such as the peripheral nerve, spinal nerve root, or spinal cord. This group included Patients 1-7

^d^ Non-neural-lesion group is defined as the group of patients with potential cause of pain not primarily originating from lesions in the neural structures. This group included Patients 8-11.

^e^ Reduction in NPRS score is defined as the difference between the preoperative and postoperative NPRS scores.

^f^ Percentage pain reduction is defined as the percentage reduction in the NPRS score after SCS.

**Table 5:** Comparison of clinical variables and pain outcomes between CRPS and non-CRPS groups

		Overall (n = 11)	CRPS vs non-CRPS					
			CRPS ^b^ (n = 5)	Non-CRPS ^c^ (n = 6)	*p-*value	Effect size for independent sample *t*-test		
						Cohen’s d	95% CI	Magnitude
Age (y), mean ± SD		45.7 ± 13.6	40.6 ± 16.2	50 ± 10.4	0.274	0.707	-0.524 to 1.938	Medium
Pain duration before SCS (m), mean ± SD		47.9 ± 29.2	55 ± 26.7	42 ± 32.2	0.491	0.435	-0.769 to 1.639	Small
Preop NPRS score, mean ± SD		9.5 ± 0.9	9.6 ± 0.5	9.3 ± 1.2	0.662	0.314	-0.881 to 1.510	Small
Postop NPRS score, mean ± SD		3.6 ± 2.7	4.8 ± 3.6	2.5 ± 1.4	0.176	0.879^a^	-0.376 to 2.133	Large
Reduction in NPRS score ^d^, mean ± SD		5.9 ± 2.8	4.8 ± 3.6	6.8 ± 1.7	0.245	0.737	-0.498 to 1.972	Medium
Percentage pain reduction (%) ^e^, mean ± SD		62.5 ± 27.8	50 ± 35.6	72.9 ± 15.6	0.187	0.867^a^	-0.386 to 2.119	Large
Follow-up (m), mean ± SD		48.6 ± 24.3	59.6 ± 20.5	39.3 ± 24.9	0.181	0.881^a^	-0.374 to 2.135	Large
						Odds ratio	95% CI	
Sex, n (%)	Male	4 (36.4)	1 (20)	3 (50)	0.546	1.000		
	Female	7 (63.6)	4 (80)	3 (50)		4.000	0.265 to 60.328	

CI, confidence interval; CRPS, complex regional pain syndrome; m, month; n, number of patients; NPRS, numeric pain rating scale; SCS, spinal cord stimulation; SD, standard deviation; y, year

^a^ Indicates a large magnitude of difference of the individual variable between both groups.

^b^ CRPS group is defined as the group of patients with a diagnosis of CRPS. This group included Patients 6, 7, and 9-11.

^c^ Non-CRPS group is defined as the group of patients without a diagnosis of CRPS. This group included Patients 1-5 and 8

^d^ Reduction in NPRS is defined as the difference between the preoperative and postoperative NPRS scores.

^e^ Percentage pain reduction is defined as the percentage reduction in the NPRS score after SCS.

Two patients (Patients 1 and 6) reported intermittent loss of stimulation related to neck positioning. One patient (Patient 2) experienced occasional ipsilateral leg stimulation. Another patient (Patient 10) developed discomfort at the implantable pulse generator (IPG) site during trunk motion, requiring IPG relocation. Another patient (Patient 5) did not achieve complete pain coverage. One individual with CRPS type 2 (Patient 7) showed worsening pain, leading to device removal; pain did not improve after removal. One patient with CRPS type 1 (Patient 11) who used a non-rechargeable IPG experienced early battery depletion at 1 year. This required IPG replacement and reprogramming to extend battery life. No device infections or electrode migrations were observed.

## Discussion

Melzack and Wall’s gate control theory, proposed in 1965, paved the way for SCS [51]. In typical circumstances, stimulation of small nociceptive fibers in peripheral tissue opens the dorsal-column gate in the spinal cord, allowing pain signals to ascend to the brain. By contrast, SCS activates large fibers, which transmit touch and vibration. This activation closes the gate on the small fibers, thus modulating pain transmission [52, 53]. Neurochemical changes within the spinal cord, triggered by SCS, also likely contribute to pain modulation [54–58]. Additionally, SCS can alleviate sympathetically mediated pain, as in CRPS type 1 [59–62], and it has been shown to enhance distal blood flow in ischemic conditions, such as myocardial ischemia or ischemia from peripheral arterial occlusive disease [63–68].

Compared with thoracolumbar SCS for failed back surgery syndrome or lower limb pain, cervical SCS has been less frequently employed for upper limb pain. Nonetheless, it can effectively relieve refractory pain caused by root avulsion injuries, CRPS, failed neck surgery syndrome, ischemic limb pain, and phantom limb pain following upper limb amputation [38, 69–77]. Another emerging indication for cervical SCS is neuropathic pain associated with syringomyelia [78–80]. Moreover, stimulation at the cervicomedullary junction or high cervical cord has been used to treat pain syndromes involving the head and face, such as trigeminal neuropathic pain, trigeminal deafferentation pain, postherpetic neuralgia, poststroke facial pain, occipital neuralgia, and severe headache disorders [81–90]. As in thoracic or lumbar SCS, the choice of cervical vertebral level depends on the spinal cord segments that correspond to the patient’s upper limb pain distribution.

In the present study, the most frequent indication for SCS was CRPS, followed by neuropathic pain from cervical root avulsion in brachial plexus injuries, intramedullary or nerve root lesions, and ischemic pain due to peripheral arterial occlusion. Our surgical outcome analysis demonstrated statistically significant pain relief in overall patients and in each subgroup. This finding underscores the importance of rigorous patient selection and trial stimulation in identifying suitable SCS candidates.

CRPS involves spontaneous or evoked regional pain that is disproportionate in timing or severity relative to any known traumatic event or lesion [91, 92]. It often arises from trauma, surgery, or limb immobilization [93–97]. Proposed mechanisms include inflammation, vasomotor instability, peripheral and central sensitization, maladaptive neuroplasticity, and immune dysregulation [5, 97–99]. Patients typically exhibit sensory disturbances, impaired voluntary motor function, vasomotor dysfunction, and autonomic imbalance [100–102]. CRPS can lead to profound disability, psychiatric sequelae, and impaired social cognition [103–107]. Approximately 10%‒20% of cases progress to a chronic, treatment-resistant state [108]. Multiple investigations support SCS as an effective therapy for refractory CRPS [109–112]. In our cohort, 4 of 5 patients with CRPS showed favorable long-term outcomes after permanent device implantation. SCS may also alleviate CRPS-related myoclonus, which affects about one-fourth of patients [113]. Moreover, novel SCS modalities, including 10 kHz high-frequency stimulation, high-density stimulation, and burst stimulation, have been introduced to augment pain control in CRPS [114–116].

Brachial plexus avulsion (BPA) injury is a debilitating traumatic condition that often leads to neuropathic pain [117–119]. This pain occurs in the majority of BPA cases [118–121] and can be difficult to treat effectively. BPA typically results when an acute traction force disrupts the C5‒T1 nerve roots of the brachial plexus [122]. Traumatic avulsion of dorsal rootlets causes hyperexcitability of nociceptive inputs in the spinal dorsal horn. This deafferented state leads to elevated levels of glutamate and other excitatory amino acids, along with increased N-methyl-D-aspartate receptor activity, and reduced inhibitory neurotransmitters such as acetylcholine, γ-aminobutyric acid, and glycine [123]. These changes can generate spontaneous high-frequency discharges and ongoing neuropathic pain [124].

Various surgical interventions are used to manage neuropathic pain in BPA. Peripheral nerve procedures (e.g., nerve repair, transfer, or neurolysis) may offer variable pain outcomes [17, 125–127] but are limited by the time since injury [128, 129] and by a variable incidence of persistent postoperative pain [130, 131]. Dorsal root entry zone lesioning is effective for deafferentation pain but is invasive and may not relieve continuous background pain [132–138]. It also carries a risk of neurological deficits and is usually reserved for intractable BPA-related pain. By contrast, SCS is a minimally invasive and reversible treatment that has shown promising results for BPA pain [73, 76, 139, 140]. At our center, we typically use dorsal root entry zone lesioning for BPA patients with no residual sensory or motor function. However, SCS is preferred for incomplete BPA injuries to preserve any remaining neurological function. Two individuals in our series (Patients 3 and 4) had BPA and underwent successful SCS implantation. Patient 3 experienced recurrent pain several years after dorsal root entry zone lesioning, and Patient 4 retained intact sensation in the painful region. Both patients achieved satisfactory long-term pain relief with SCS.

Peripheral neuropathy commonly presents with sensory disturbances, such as numbness, paresthesia, muscle weakness, and neuropathic pain. Its etiologies include trauma, surgery, tumors, infections, diabetic peripheral neuropathy, postherpetic neuralgia, chemotherapy-induced neuropathy, and chronic idiopathic axonal polyneuropathy [141–147]. SCS is an effective therapy for painful peripheral neuropathy from various causes [148–151]. In our series, 1 patient (Patient 5) with painful neuropathy following resection of a cervical nerve root schwannoma achieved a 70% pain reduction after SCS.

Central neuropathic pain from intramedullary spinal cord lesions (e.g., spinal cord injury, neoplasms, and syringomyelia) is especially challenging to treat. Its pathophysiology involves structural and functional central nervous system changes, aberrant pain signal conduction, and inflammatory pathways. Alterations in voltage-gated sodium channel and N-methyl-D-aspartate receptor expression, along with microglial activation, contribute to persistent central neuropathic pain [152]. In our study, 2 patients (Patients 1 and 2) experienced intractable neuropathic pain due to intramedullary lesions. Patient 1 developed neuropathic pain after removal of a cervical cord ependymoma. Patient 2, who had Chiari I malformation and cervical syringomyelia, continued to experience left upper extremity neuropathic pain despite surgical decompression of the foramen magnum. Both patients obtained meaningful pain relief following SCS.

Central neuropathic pain is a distinct clinical manifestation that frequently accompanies syringomyelia [153–156]. Approximately 60% of patients with syringomyelia develop neuropathic pain [157]. Even when foramen magnum decompression successfully reduces the syrinx cavity, pain may persist. Research on SCS for neuropathic pain relief in Chiari I malformation with syringomyelia is limited [158, 159], and its efficacy following decompression remains unclear.

In our trial stimulation, 3 patients with Chiari I malformation and syringomyelia underwent conventional SCS. Only 1 (Patient 2) responded favorably and proceeded to device implantation, whereas the remaining 2 did not achieve adequate pain relief. In a single case report, Yamana et al. described successful neuropathic pain relief in a patient with syringomyelia related to Chiari I malformation using fast-acting sub-perception therapy [159]. Further studies are needed to clarify whether conventional SCS or alternative stimulation protocols can consistently mitigate syringomyelia-related neuropathic pain.

**Fig. 4:**
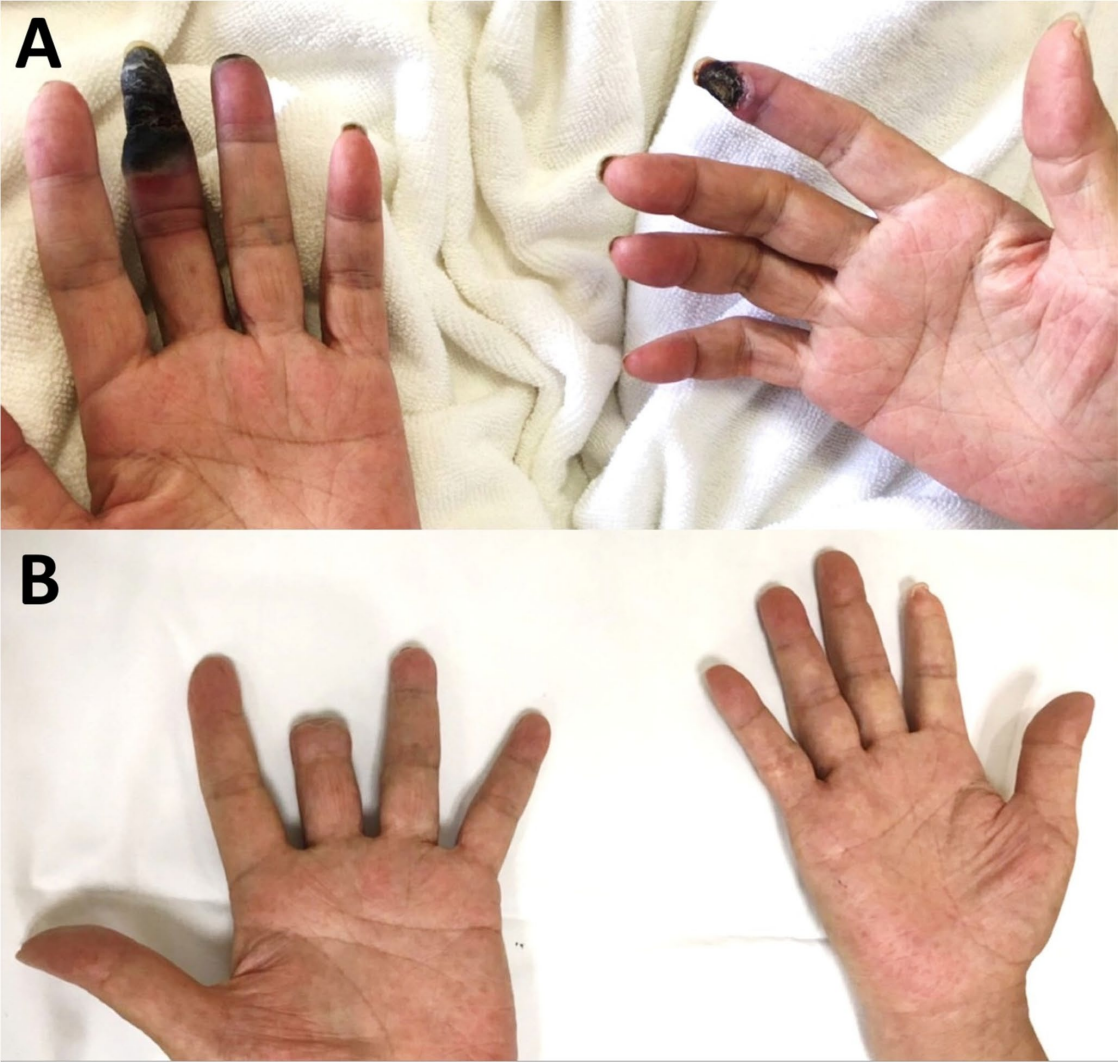
Clinical response in Patient 8 (eosinophilic granulomatosis with polyangiitis). **(A)** Preoperative photograph showing bilateral digital gangrene and ischemic discoloration. **(B)** Few months after cervical SCS: complete pain relief, restored distal perfusion, and auto-amputation of necrotic fingertips, allowing functional hand use

**Fig. 5:**
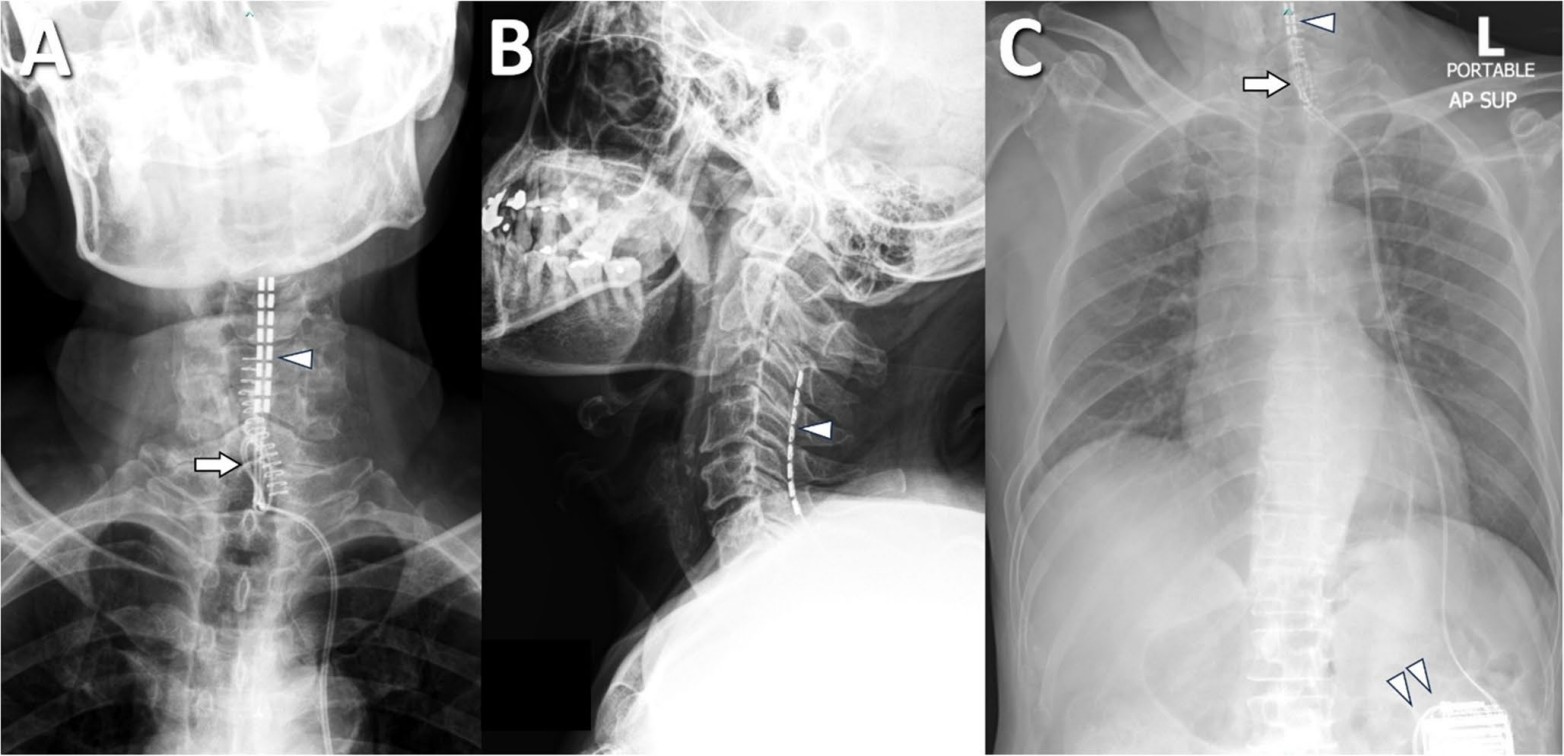
Permanent paddle-lead system in Patient 8. **(A)** Cervical AP radiograph: 2 × 8 paddle lead (arrowhead) with a tension-relieving loop (arrow). **(B)** Lateral radiograph confirming posterior placement of the paddle (arrowhead). **(C)** Chest radiograph: paddle lead (single arrowhead), strain-relief loop (arrow), and implantable pulse generator (double arrowheads) in the left flank

Ischemic limb pain represents a primary symptom in various diseases that severely impair blood flow to distal extremities, often with visible ischemic changes. SCS is an option for advanced-stage ischemic vascular conditions in which medical treatment fails or patients cannot undergo endovascular or surgical revascularization [160–163]. The proposed mechanisms include SCS-mediated reduction of sympathetic vasoconstriction, release of endothelium-derived nitric oxide, improved microcirculation, reduced inflammation, and enhanced wound healing [162, 164–166]. At our center, we have employed SCS for patients with inoperable peripheral vascular disease, yielding significant pain relief and improved distal circulation. In this study population, only 1 individual (Patient 8) with eosinophilic granulomatosis with polyangiitis (Churg-Strauss syndrome) received cervical SCS for digital arterial occlusion. Postoperatively, distal blood flow returned to normal, and ischemic pain completely resolved (Fig. 4, 5).

Accurate epidural electrode placement is crucial for successful SCS. In patients without prior spinal operations, percutaneous insertion typically poses no difficulties. By contrast, post-laminectomy changes or previous spinal cord surgery often lead to epidural scar tissue or fibrosis, which can obstruct electrode passage and reduce stimulation efficacy [167, 168]. Such fibrosis may also provoke compressive myelopathy following SCS [169–172]. We have encountered this challenge particularly with percutaneous trial electrodes, where the lead could not traverse fibrotic areas. After failing the percutaneous approach, we proceeded with open surgery to remove as much epidural granulation or fibrotic tissue as possible while preserving the dura mater. Thickened fibrosis can otherwise block electrical transmission to the dorsal columns and render trial stimulation ineffective. In post-laminectomy patients who lack intact laminae, we recommend suturing the electrode to the dura mater to prevent displacement.

If trial stimulation is successful, the next operation involves removing the trial electrode and placing a permanent one. In patients without spinal lamina, we generally use a paddle lead and create an artificial lamina with mini‒titanium plates. These plates, fixed with screws to the residual lateral bone, are contoured to cover and stabilize the paddle electrode. Close contact between the electrode surface and dura mater improves electrical transmission to the dorsal column of the spinal cord. During plate insertion, excessive pressure on the paddle lead should be avoided to prevent cord compression.

In our series, 3 patients experienced adverse effects specific to cervical SCS, which we had not observed in thoracolumbar SCS cases. Patients 1 and 6 had intermittent loss of stimulation, and Patient 2 occasionally reported unwanted ipsilateral leg stimulation. All 3 adverse effects occurred only when the patients’ necks were in certain positions, and stimulation returned to baseline once their necks were moved back to a more typical posture. The high mobility of the cervical spine may temporarily alter the electrode contact or current pathway, causing variations in stimulation efficacy. These complications persisted despite secure electrode anchoring and optimal parameter adjustments.

Previous studies have noted that changes in body position can vary SCS amplitude and coverage, particularly in the cervical region [34]. In some instances, patients must maintain uncomfortable postures to preserve consistent stimulation, potentially limiting the device’s overall effectiveness.

Another major concern in cervical SCS is the risk of iatrogenic neurological injury. Because the spinal cord enlargement extends from the C3 to C7 vertebral levels, degenerative changes can narrow the spinal canal, leaving a shallow cerebrospinal fluid space around the cord and nerve roots [34]. These anatomical constraints may place patients at higher risk of neurologic injury than in thoracic or lumbar SCS. A large series investigating complications in cervical SCS reported a 0.5% rate of spinal cord injury, and rates of 1.1%, 1.4%, and 11.7% for other neurological, medical, and general perioperative complications, respectively. In that series, patients with preexisting cervical spondylotic myelopathy or cervical spinal stenosis were more likely to develop complications [173].

Because cervical SCS for upper limb pain is relatively uncommon compared with thoracolumbar SCS for back or lower limb pain, our study is limited by the small sample size and scant number of patients for each pain etiology. In addition, the heterogeneity of etiologies treated by cervical SCS in our series precludes drawing definitive conclusions about its efficacy for any specific cause of refractory upper extremity pain. Single-center experience may result in a lack of generalizability and difficulty with reproducible procedures in other medical centers. Retrospective design could be associated with incomplete data in some aspects. Selection bias was a limitation; only SCS trial responders who underwent device implantation were recruited into the study. Furthermore, our series evaluated neither standardized functional nor quality-of-life outcomes. Clinical assessment of these facets is advocated in future research.

## Conclusions

The present study demonstrated that SCS effectively alleviates intractable upper limb pain arising from diverse etiologies, including CRPS, brachial plexus avulsion, postsurgical cervical root and cord lesions, syringomyelia, and digital arterial occlusion. Satisfactory outcomes depend on careful patient selection and rigorous trial stimulation. Nonetheless, some patients may experience diminished analgesic effects over several years, underscoring the importance of long-term follow-up to sustain the therapeutic benefits of SCS.

## Data Availability

No datasets were generated or analysed during the current study.
